# Artemisinin Derivatives and Synthetic Trioxane Trigger Apoptotic Cell Death in Asexual Stages of *Plasmodium*

**DOI:** 10.3389/fcimb.2018.00256

**Published:** 2018-07-26

**Authors:** Sarika Gunjan, Tanuj Sharma, Kanchan Yadav, Bhavana S. Chauhan, Sunil K. Singh, Mohammad I. Siddiqi, Renu Tripathi

**Affiliations:** ^1^Academy of Scientific and Innovative Research, New Delhi, India; ^2^Division of Parasitology, Central Drug Research Institute (CDRI), Council of Scientific and Industrial Research (CSIR), Lucknow, India; ^3^Division of Molecular & Structural Biology, Central Drug Research Institute (CDRI), Council of Scientific and Industrial Research (CSIR), Lucknow, India

**Keywords:** arteether, artesunate, CDRI-97/78, apoptosis, *Plasmodium*

## Abstract

Although over the last 15 years, prevalence of malaria became reduced by over half but developing resistance against artemisinin derivatives and its combinations, which are only ray of hope to treat resistant malaria set back the control efforts and the key hinderence to achieve the goal of malaria elimination till 2030. In spite these artemisinins are precious antimalarials, their action mechanism is yet to be fully understood. Reactive oxygen species (ROS) produces by cleavage of endoperoxide bridge of artemisinin derivatives are known to be its antimalarial efficacy. Since ROS could induce apoptosis, here we had explored the effect of artemisinin derivatives on apoptotic machinery of malaria parasite, *Plasmodium falciparum* and its survival. We have studied the effect of a/β arteether, artesunate and a synthetic 1, 2, 4 trioxane on mitochondria, caspase activity and DNA during asexual blood stages of *Plasmodium falciparum* 3D7. Results have shown that cleavage of peroxide bridge of artemisinin derivatives and 1,2,4 trioxane generate reactive oxygen species which depolarize mitochondrial membrane potential and make it permeable which further followed by activation of caspase like enzyme and DNA fragmentation, which are hallmark of apoptotic cell death. These findings suggest that artemisinin derivatives and synthetic trioxane induce apoptosis like phenomena in erythrocytic stage of malaria parasite; *Plasmodium falciparum*.

## Introduction

Lots of efforts are carrying out to control the deadly disease, malaria but it is still a burden to human health and ~3.2 billion people are at risk of malaria word wide. According to WHO 445,000 deaths and 216 million malaria cases were reported in 2015 but due to employment of artemisinin and drug impregnated bed nets, 30% decrease in malaria cases and 47% in mortality rate was observed since 2000 (White et al., 2014; WHO, 2014, 2015). In spite of having these magic drugs, the decreasing clinical efficacy and emerging resistance to artemisinin derivatives in Thailand and Cambodia have raised an alarming situation about future treatment options (Price et al., 1996; Amaratunga et al., 2012; Phyo et al., 2012; Ferreira et al., 2013). Peroxide bridge linkage of artemisinin, a sesquiterpene trioxane lactone, is known to be responsible for its anti-malarial activity (Butler, 1992). Since artemisinin is poorly soluble in water as well as in oil, it becomes reduced into dihydroartemisinin (DHA) and its derivatives such as the water-soluble artesunate and oil-soluble artemether and arteether. Due to limited availability of artemisinins, continued efforts led to the development of synthetic trioxanes. A novel trioxane 97/78, developed by CSIR-Central Drug Research Institute (CDRI), India, has shown promising antimalarial activity and is currently under clinical trials (Singh et al., 2011). CDRI-97/78 contains 1, 2, 4-trioxane nucleus similar to endoperoxide lactone of artemisinin. It emerged as lead compound with excellent pharmacological antimalarial activity (Singh et al., 2004, 2011; Griesbeck et al., 2005). Although, at the present time artemisinin derivatives are the main stay of malaria therapy, its mode of action is a topic of debate. Considering its importance, here we explored the action mechanisms of artemisinin derivatives (ART, ARS) and a CDRI compound- 97/78 during erythrocytic cycle of parasites via apoptotic markers as apoptosis found to be a novel cell death pathway in Plasmodium and multiple markers of apoptosis have been observed in different stages of Plasmodium life cycle that occur in vector and the host (Al-Olayan et al., 2002; Meslin et al., 2007; Ch'ng et al., 2010; Gunjan et al., 2016).

## Materials and methods

### *In-vitro* cultivation of *P. falciparum*

*In vitro* culture of chloroquine sensitive strain (*Pf* 3D7) of *P. falciparum* was carried out in fresh human erythrocytes at 5% hematocrit in complete RPMI-1640 (HEPES modified) medium (Sigma) supplemented with 0.5% AlbuMaxII, 0.2% glucose, 0.2% NaHCO_3_ and 15 μM hypoxanthine and incubated at 37°C in CO_2_ incubator (Trager and Jensen, 1976). Parasite growth rate and stage was determined by the examination of Giemsa's stained thin blood smears of infected erythrocytes.

### Evaluation of *in vitro* antimalarial profile of drugs

To evaluate antimalarial activity of drugs on erythrocytic stages of the *P. falciparum* 3D7, SYBRGreen I fluorometric assay was carried out with some modifications (Johnson et al., 2007). Briefly, two fold serial dilutions of drugs were prepared in 96 well plates and then 50 μl asynchronous culture (~95% ring) of infected erythrocytes with 0.8–1% parasitaemia and 1% hematocrit was added to each well (100 μl-final volume). Eight wells were treated as positive control (without drug) and 4 wells as negative controls (without parasite and drug). Further culture were incubated at 37°C for 72 h in CO_2_ incubator. After 72 h, 100 μl of lytic buffer containing 1X SYBR Green was added to each well and incubated for 2 h at room temperature in dark. Fluorescence of SYBR Green was recorded using fluorescence reader at Ex. 485 nm, Em. 535 nm. IC_50_ was calculated on the basis of DNA content of the parasite by using MS-Excel template.

### Computational studies

Considering that the metacaspase protein (PLASMODB id - PF3D7_1354800) may be potential drug target, we attempted 3D-structural investigation on sequenced protein of *P. falciparum*. Modeled protein was selected using methodology described previously from our lab (Gunjan et al., 2016). Structure of arteether, artesunate and parent compound CDRI 97/78 used for docking studies were built and minimized using Gaussian version 09 (Dennington et al., 2009; Frisch et al., 2009). Computational studies were performed on the metacaspase protein model to get the molecular insights of how the drugs were bound to its target protein. Binding site was generated using the SiteMap module of Schrodinger package (Halgren, 2009). Further, the grid around binding sites identified by SiteMap were generated using Grid generating module of Schrodinger software package (Schrödinger, 2016b). Van der Waals radius used for scaling was 1.0 Å and default settings were used for other parameters. Both protein and ligands (arteether, artesunate, compound CDRI 97/78) were prepared using the Ligand and Protein preparation modules of Schrodinger software package (Schrödinger, 2016a,c). Prepared ligands were then docked with Ubiquitin Proteasome using Glide-7.1 module (Friesner et al., 2006). Docking was performed using Extra precision mode and default setting were used for docking. Ligand receptor interaction images were generated using the “Ligand interaction” module of Schrodinger software package. Poses for interaction studies were selected from largest cluster. The interactions which were analyzed in this module were “Hydrogen Bonding, Pi-Pi interaction, Pi-Cation interaction, Salt Bridges” at cut-off radius of 2.50 Å. Coulomb surface for model and ribbon view were generated using UCSF-Chimera version 1.10 (Pettersen et al., 2004).

### Caspase assay

To measure the caspase-like protease activity during erythrocytic stage in drug treated and untreated parasites EnzChek® Caspase-3 Assay Kit (Molecular probe) was used and assay was performed according to the manufacturer's instructions with some modification. In brief, infected erythrocytes (iRBCs) having 8–10% parasitaemia were exposed with 10 nM of ART/ARS and 100 nM of CDRI-97/78 for 24 h. Cell free parasites were prepared by saponin lysis of drug treated and untreated iRBCs. Parasite lysate were prepared by mild sonication and 2X reaction buffer containing caspase substrate Z-DEVD–R110 was added into 1:1 ratio followed by incubation at room (25°C) temperature for 30 min. in dark (Gunjan et al., 2016). Increase in fluorescence caused by cleavage of the Z-DEVD-R110 substrate was measured at excitation and emission wavelengths of 485/20 and 530/20 nm, respectively using micro plate reader (Synergy HT Biotek). In a parallel set of reactions, the caspase inhibitor Ac-DEVD-CHO was added to the reaction mixture before the addition of parasite lysate. All the experiments were performed in duplicate.

### Mitochondrial membrane potential (ΔΨm)

To determine the effect of ART, ARS, and CDRI-97/78 on the mitochondria of erythrocytic stages of *P. falciparum*, iRBCs were exposed with these drugs for 6 and 24 h and further mature stage enriched parasitized red blood cells were stained with 6 μM JC-1 dye (Sigma-Aldrich) for 20 min. in dark at 37°C. Cells were washed twice and resuspend in 0.5 ml assay buffer for acquiring the fluorescence using flow cytometer.

Acquisition was carried out using FACS-CELLQUEST software on FACS Calibur (BD Biosciences) and analysis of flow cytometeric data was performed using Flow Jo software (Tree star Inc., Ashland, OR). Dissipation in ΔΨm was measured by calculating the ratio between red and green fluorescence (i.e., 590/530 nm) (Gunjan et al., 2016).

### *In situ* detection of DNA fragmentation by terminal deoxynucleotidyl transferase (TdT)-mediated dUTP nick end labeling (TUNEL)

DNA fragmentation in malaria parasite during erythrocytic cycle was analyzed using an *in situ* cell death detection kit (Promega). Briefly, asexual stages of *P. falciparum* were treated with ART (10 nM), ARS (10 nM), and CDRI-97/78 (100 nM) for 24 h followed by saponin enrichment to isolate cell free parasite. Parasites were fixed with 1% paraformaldehyde for 1 h at 4°C followed by permeabilization with a solution of 0.2% Triton X-100. Fixed and permeabilized parasites were labeled with the TUNEL solution for 1 h at 37°C. Reactions were terminated by adding 20 nM EDTA. Finally cells were resuspended in PBS and analyzed by LSRII flow cytometer (BD Biosciences) equipped with 488 nm argon laser (Gunjan et al., 2016). Percentage of TUNEL positive cells were calculated using Flow Jo analysis software.

### Measurement of ROS level in blood stages of *P. falciparum*

Intracellular ROS levels were measured in treated and untreated cells as described previously (Gunjan et al., 2016). In Brief, iRBCs having 8–10% parasitaemia were exposed with 10 nM ART and ARS,100 nM of CDRI-97/78 for 24 and 48 h. Further cells were incubated with 10 μM concentration of dichlorodihydrofluorescein diacetate (H_2_DCFDA) (Sigma) and incubated in CO_2_ incubator at 37°C for 30 min in dark. Samples were washed twice with 1X PBS and cell free parasites were isolated by saponin lysis of iRBCs (Gunjan et al., 2016) and resuspended in PBS for acquiring fluorescence of DCF. Acquisitions were done on FACS caliber flow cytometer and data were analyzed using FlowJo 8.1.0 software (Tree Star Inc., USA).

### Statistical analysis

The statistical analyses of the antimalarial effect of drugs on the asexual stages of *P. falciparum* were performed using Student's *t*-test. The statistical analyses of other apoptotic markers were performed using analysis of variance (ANOVA) and the Tukey *post hoc* test.

## Results

### *In vitro* antimalarial profile of α/β arteether (ART), artesunate (ARS) and CDRI-97/78

**Figure 1:**
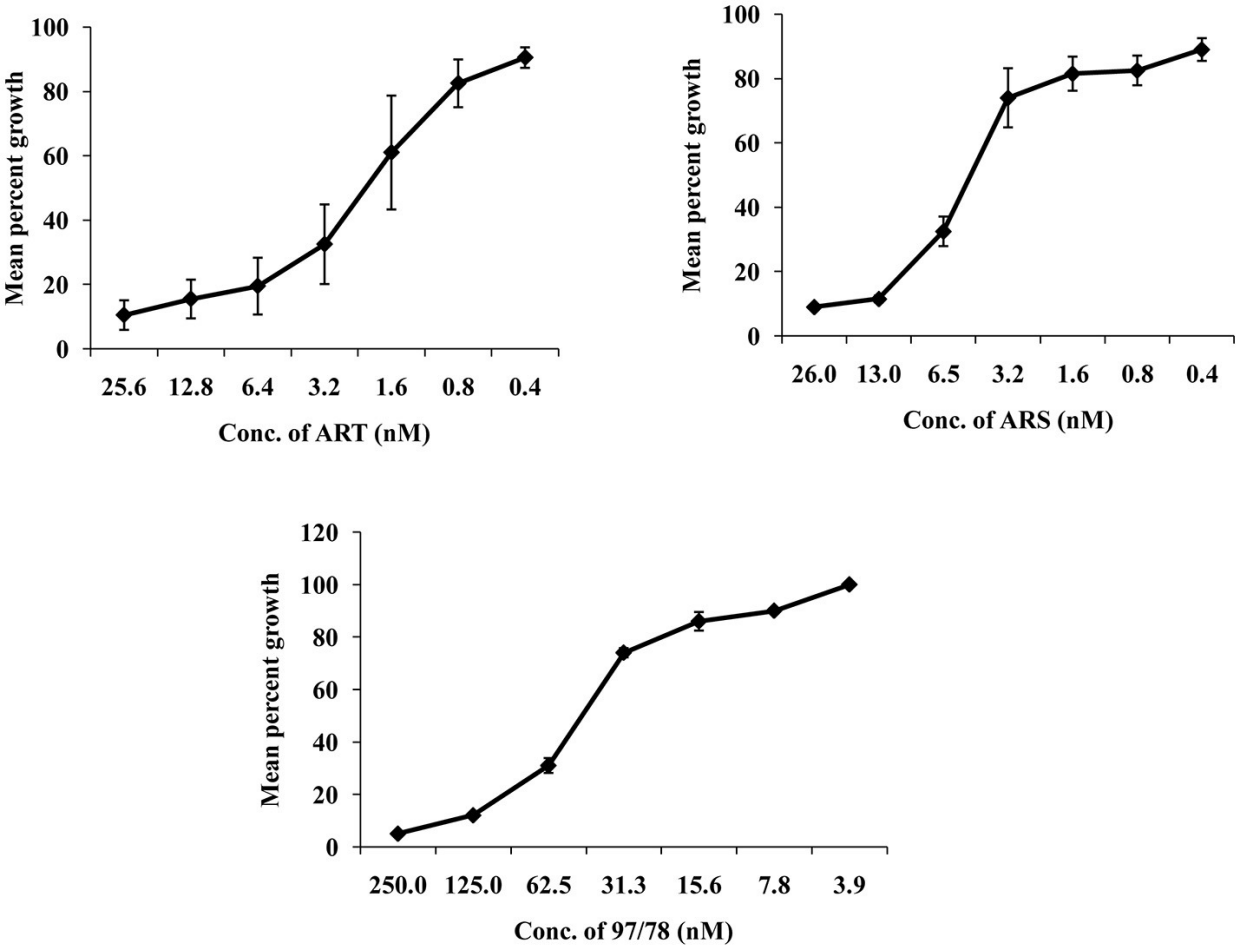
Typical dose response of ART, ARS, and CDRI-97/78 on growth of CQ sensitive strain of *P. falciparum*. **(A)** Graph showing ~90 and 50% inhibition in parasite growth at 25.6 and 2.19 nM conc. of ART respectively. **(B)** ~90 and 50% parasite growth inhibition is shown at 26 and 4.79 nM of ARS respectively. **(C)** CDRI synthetic compound 97/78 inhibits ~90 and 50% growth of *Plasmodium* at 125 and 49 nM.

*In vitro* growth of *P. falciparum* 3D7 was inhibited by ART,ARS and CDRI-97/78 in a dose dependent manner. The IC_50_ values of ART, ARS and CDRI-97/78 were found to be 2.19 ± 0.9 nM, 4.79 ± 0.7 nM and 49 ± 2.8 nM (Figure 1). For further studies to check the effect of these drugs on apoptotic markers; mitochondrial outer membrane potential, caspase like activity and DNA fragmentation ~IC_90_ concentration of drugs was used.

### Computational studies

**Figure 2:**
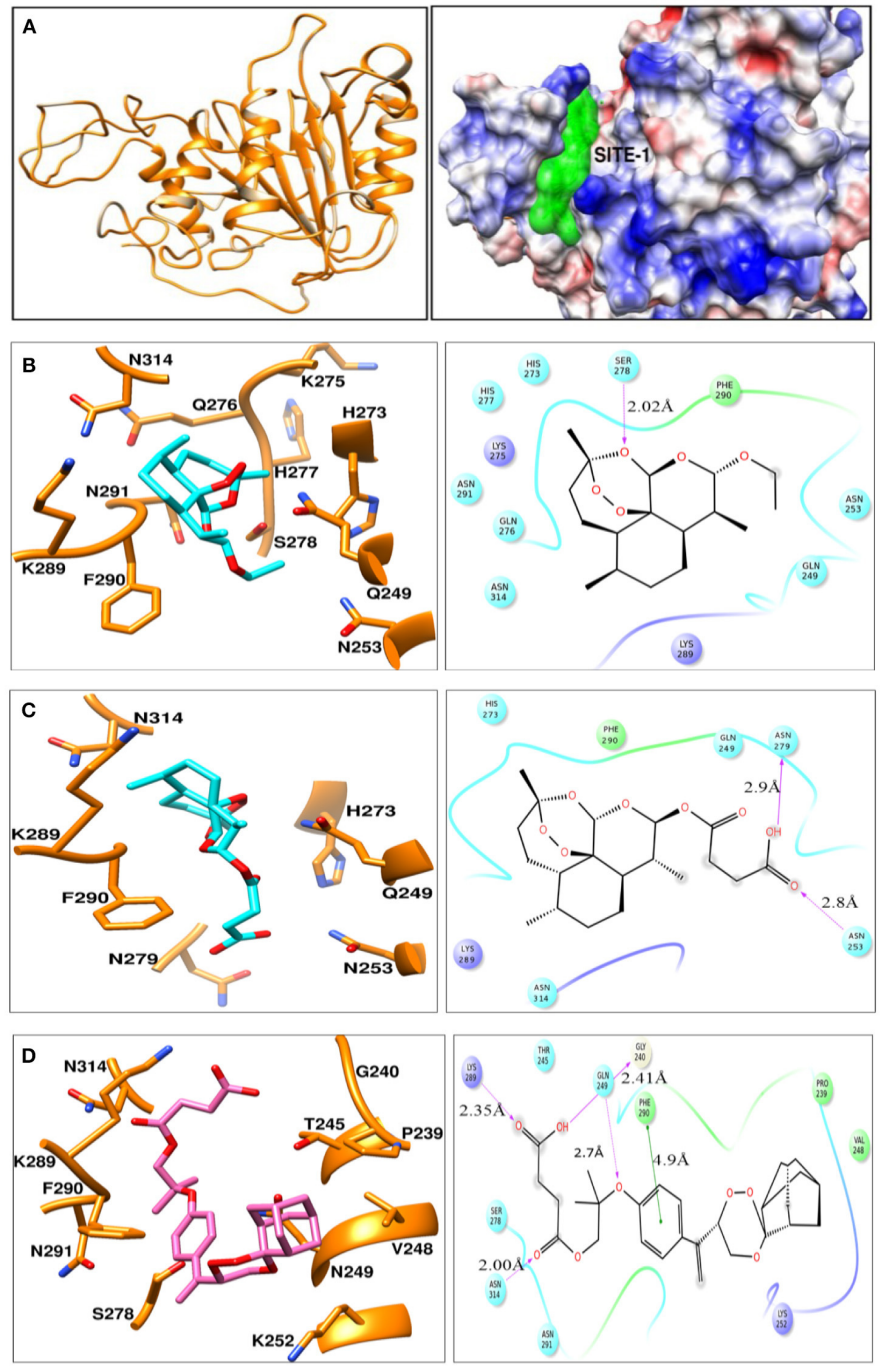
**(A)** Ribbon view model of metacaspase modeled protein. Indicates coulomb surface model of receptor along with the best predicted binding site (green color) by SiteMap module of Schrodinger version 2016-2. **(B–D)** indicates the ligand-interaction plots of ART, ARS, and compound CDRI- 97/78 respectively along with interacting residues of Metacaspase protein identified using ligand interaction module of Schrodinger-2016-2. The color code of various interacting residues indicates their physiochemical property. Blue stands for polar, red for charged negative, purple for charged positive, green for hydrophobic. Light shadows indicate solvent exposed region.

Homology models were generated using structure of the yeast metacaspase (YCA1) having PDB id - 4F6O (Wong et al., 2012). Since, for our protein MCA-1 in *P. falciparum* we were unable to retrieve any template having higher identity more than 50%. Literature review does suggest that template structure having identity greater than 30% can be utilized for homology modeling (Xiang, 2006). Thus, homology modeling was performed using template having 42% identity, 62% similarity. Ramachandran plot analysis of best model indicated 82.8% residues in favored region, 16.2% region in addition allowed region and 1.0% residues in disallowed region. Modeled protein indicated presence of binding sites, as predicted by SiteMap (Figure 2A). Of these, top 3 binding sites were used for generating grid and docking was performed around it using GLIDE-7.1. Best pose of ART, ARS, CDRI-97/98 parent compound indicated Glide Score of −6.29, −4.02, −5.36 Kcal/mol respectively. These scores were in agreement with the wet lab experimental data on Metacaspase assay. For Arteether, protein residues found important for interaction were Q249, N253, H273, K275, Q276, H277, S278, K289, F290, N291, N314 (Figure 2B). Of these most prominent interaction was hydrogen bonding of 2.02 Å with Ser278. Similarly for Artesunate, residues important for interaction were Q249, N253, H273, N279, K289, F290, and N314 (Figure 2C) residues important for interaction were Asn253 having hydrogen bond of 2.8 Å and Asn279 having hydrogen bond of 2.9 Å. For compound 97/78, important residues for interaction were P239, G240, T245, V248, Q249, K252, S278, N279, K289, F290, N291, N314 (Figure 2D). Residues important for interaction were Phe290 having 4.9 Å Pi-Pi stacking interaction, Gln249 having 2.7 Å hydrogen bond, Gly240 having 2.41 Å hydrogen bond, Lys289 having 2.35 Å hydrogen bond and Asn314 having 2.00 Å hydrogen bond (Table S1). For arteether and artesunate the binding of cyclo-hexadecane group was having same orientation but variation in binding energies was observed owing to the different ethoxy- and butanoic acid- side chains respectively. This group was mimicked by the phenoxy-propoxy moiety in compound 97/78. Residues namely Gln249, Ser278, Lys289, Phe290, and Asn314 were found to be important for interaction with compounds. Of these Gln249, His273, and Lys289 have been previously characterized for interaction with the metacaspase protein (Gunjan et al., 2016). They can be further utilized for the rational design and synthesis of anti-malarial compounds.

### Artemisinin derivatives activate caspase like enzyme in malaria parasites

**Figure 3:**
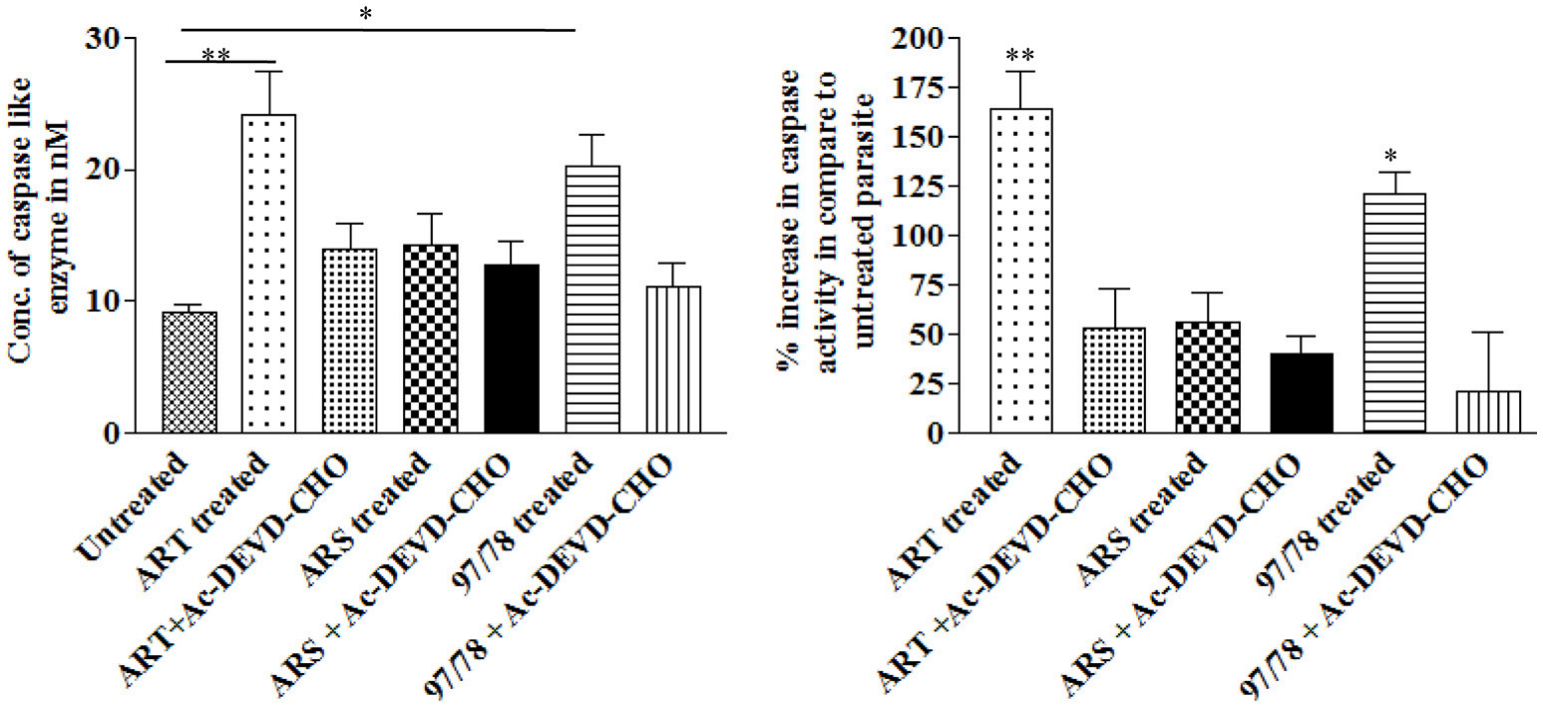
Effect of artemisinin derivatives and CDRI-97/78 on caspase like activity. Caspase-3-like activity was measured in the cytosolic fraction (100 μg protein) from untreated and drug treated parasites. ^*^p < 0.05; ^**^p < 0.01.

DEVD-specific protease activities (Caspase-3) in drug treated and untreated parasites was measured using rhodamine 110 bis-N-CBZ-L-aspartyl-L-glutamyl-L-valyl- L-aspartic acid amide—(Z-DEVD-R110). Upon enzymatic cleavage, the non-fluorescent bisamide substrate is converted to fluorescent monoamide Cytosolic fraction of artemisinin derivatives and synthesized 1,2,4 trioxane treated parasites has showed increased caspase 3 activity as compared to control viz. 164, 56, and 121% after 24 h treatment of 10 nM ART, 10 nM ARS, and 100 nM CDRI-97/78 respectively (Figure 3). Moreover, the activity of caspase-3 was significantly inhibited in the presence of Ac-DEVD-CHO, a potent inhibitor of caspase-3, indicating that *P. falciparum* contains a protease having caspase-3-like activity.

### Artemisinin derivatives reduces mitochondrial membrane potential of malaria parasite

Mitochondrial membrane potential (ΔΨm), which is an index of mitochondrial function, was also examined to evaluate the effect of artemisinin derivatives and 1,2,4 trioxane (CDRI-97/78) on mitochondria of asexual blood stages of Plasmodium. The detection of mitochondrial membrane potential in drug treated/untreated parasites was done using JC-1, cationic probe which aggregate in mitochondria due to electronegative environment inside the mitochondria and give red color fluorescence but at depolarization of mitochondrial membrane (low mitochondrial membrane potential) JC-I remains in monomeric form and give green color fluorescence.

Dot plot analysis showed that after 6 h treatment there is no significant changes in green fluorescence intensity in comparison to untreated parasites whereas after 24 h treatment, percentage of JC-1 monomer were increased significantly.

**Figure 4:**
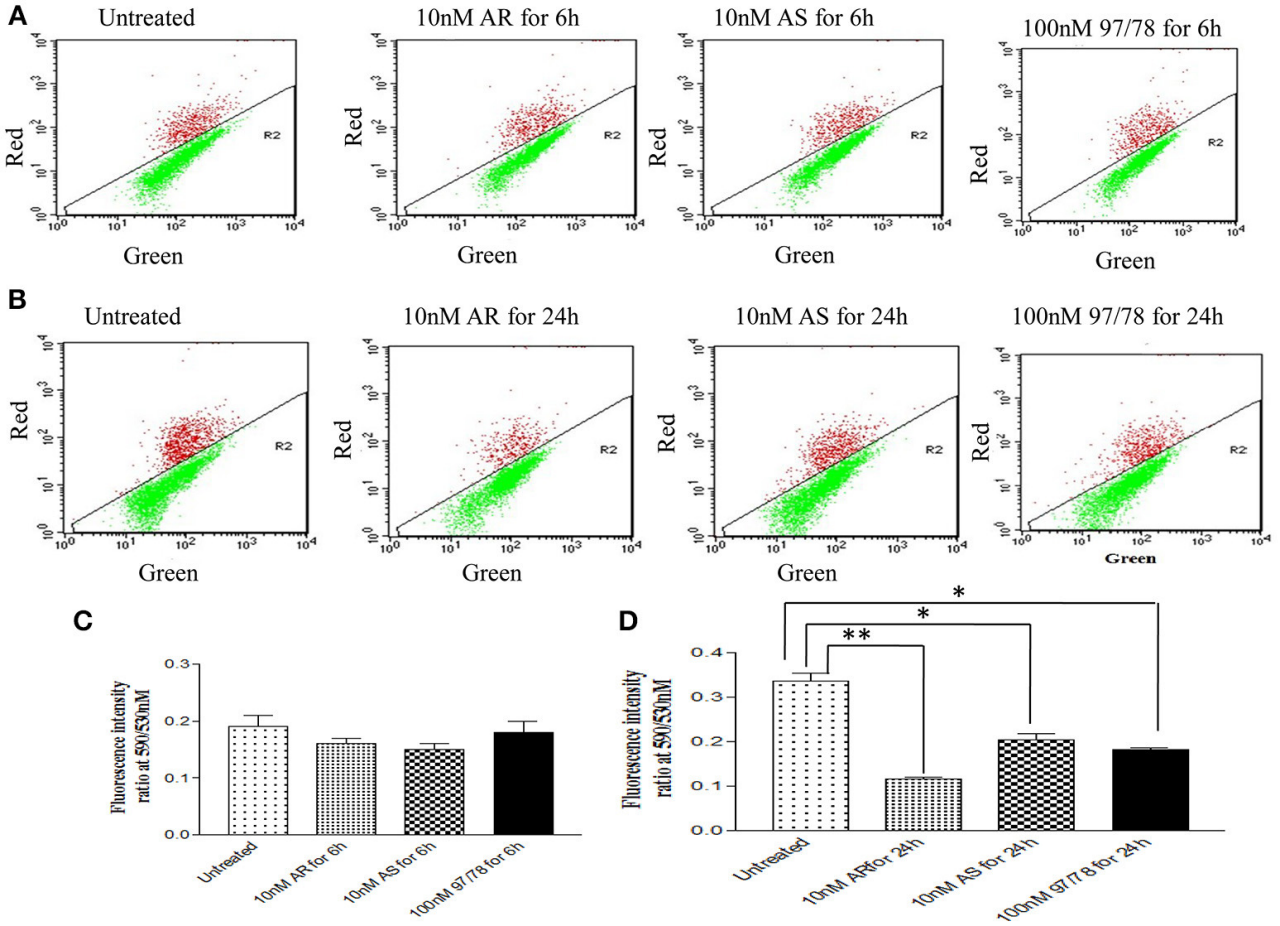
JC-1 staining of iRBCs shows reduction in mitochondrial membrane potential (ΔΨm) in *Plasmodium falciparum* parasites treated with drugs. Flow cytometry dot plot showing the gating of JC1 (red)-aggregates and JC1 (green)-monomer populations. Ratio of which represents the JC1-positive population. **(A,C)** Parasites were treated with drugs for 6 h **(B,D)** parasites were treated with drugs for 24 h. ^*^p < 0.05; ^**^p < 0.01.

In dot plot analysis, green fluorescence of JC-1 monomer was measured in R2 gate (low mitochondrial membrane potential). In untreated parasite, R2 gate contained 76% cells whereas ART, ARS and 97/78 treated parasite have shown 90, 81, and 85% cells respectively in R2 gate (Figure 4).

Red/Green ratios were obtained to determine the extent of mitochondrial dysregulation. In untreated cells red: green ratio is 0.33 ± 0.01 while it is found to be decreased to 0.11 ± 0.005, 0.20 ± 0.01 and 0.18 ± 0.004 after 24 h treatment of ART, ARS and 97/78 respectively (Figure 4).

### Increase in *in situ* DNA fragmentation in erythrocytic stage of plasmodium under artemisinin derivatives treatment

**Figure 5:**
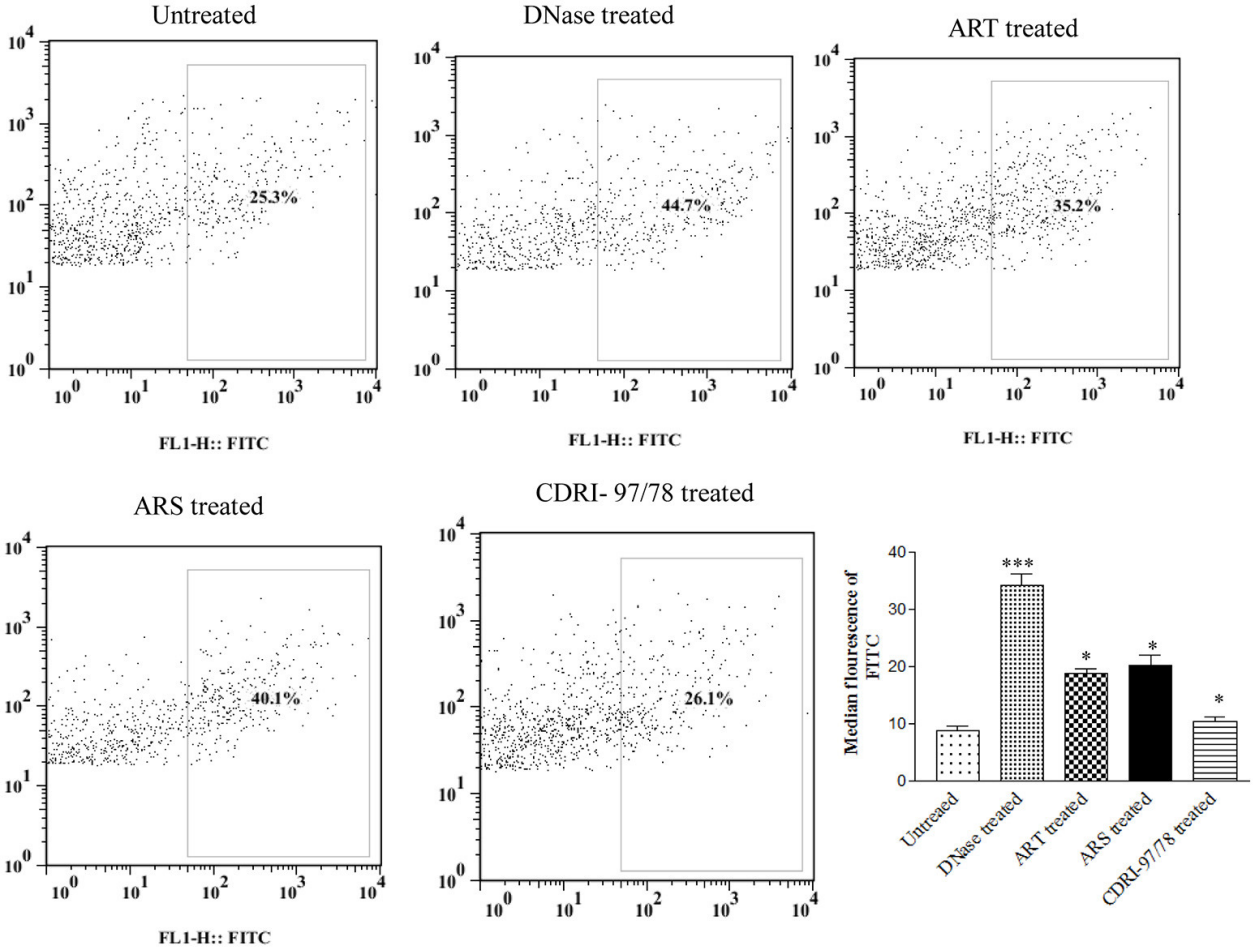
Evaluation of DNA fragmentation using TUNEL assay. Flow cytometry dot plot showing shifting of population toward FITC channel (R2 gated), and percent increase in DNA fragmentation after treatment of ART, ARS and 97/78 treated cells. Data is representative of duplicate sample of two separate experiments. *p*-value is < 0.001 for untreated vs. DNase, for ARS treated parasites, *p*-value is < 0.01 whereas for ART and 97/78 treated parasites *p*-value is < 0.05. ^*^p < 0.05; ^***^p < 0.001.

To evaluate the effect of artemisinin derivatives on DNA fragmentation in Plasmodium, parasites were exposed to 10 nM conc. of ART/ARS and 100 nM of 97/78 for 24 h. Before TUNEL staining, parasites were isolated by saponin lysis of iRBCs so that fluorescence of the parasite itself could be clearly recorded. Dot plot analysis has showed increase DNA fragmentation in these artemisinin derivatives and CDRI-97/78 treated parasites as compared to untreated (Figure 5).

### Assessment of ROS generation in artemisinin derivative and CDRI-97/78 treated/untreated parasites

**Figure 6:**
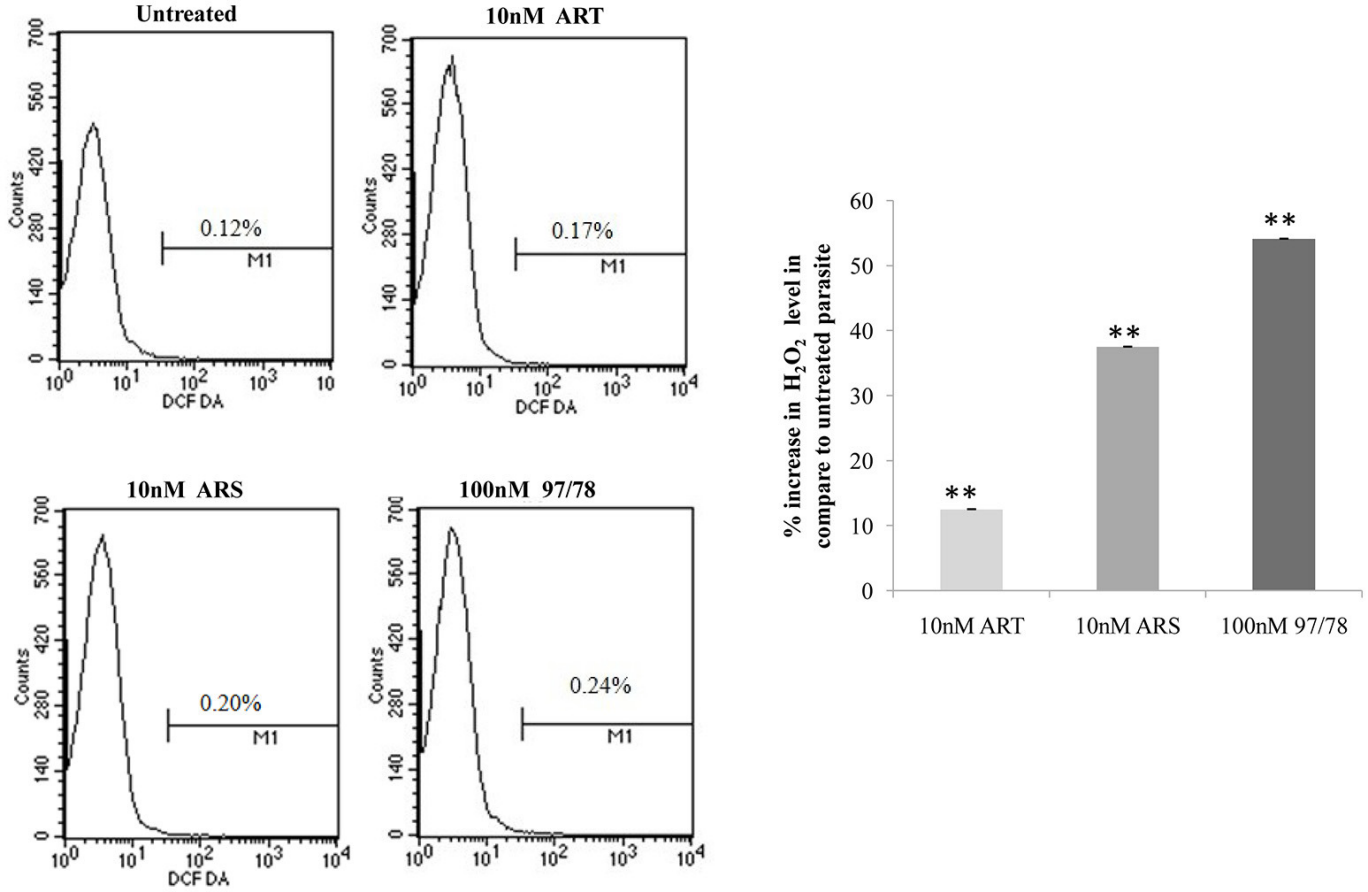
Flow cytometeric analysis of ROS generation in *P. falciparum* 3D7. Parasites isolated from all groups by saponin enrichment and loaded with DCFDA probe to evaluate ROS generation. Flow cytometry histograms showing increase in DCF positive cells after treatment with 10 nM concentrations of ART/ARS and 100 nM of CDRI-97/78 compound. Bar graph is showing percent increase of H_2_O_2_ level in drug treated parasite as compared to untreated. The experiments were performed twice (*n* = 2) and data expressed as mean values ± SEM. ^**^P ≤ 0.01 vs. control: Tukey's test.

Intracellular ROS production was measured by staining with DCFDA in treated and untreated parasites. Results showed 12.5, 37.5, and 54.1% increase in reactive oxygen species in ART, ARS, and 97/78 treated parasite respectively as compared to untreated parasites (Figure 6).

## Discussion

Artemisinin derivatives are known to be effective against both erythrocytic stages; asexual schizontocidal as well as sexual gametocytocidal and showing promise as antimalarial drugs with transmission-blocking potential (Kumar and Zheng, 1990; Price et al., 1996; Haynes and Krishna, 2004). Understanding the mode of action for artemisinin derivatives is very important to select their partner drugs and to overcome the problem of resistance toward these drugs. Even though many cellular targets have been identified, the mechanism of action of artemisinin derivatives still remains vague. No single mechanism exists for artemisinins' action and it was suggested that it functions through multiple modes (Pandey and Pandey-Rai, 2016). A decade ago, *Pf* ATP6, the *P. falciparum* sarco-endoplasmic reticulum calcium ATPase (SERCA) was identified as potential target for artemisinin (Friesner et al., 2006). It has been proposed that the endoperoxide bridge is responsible for antimalarial activity as it generates free radical which alkylates the different parasitic protein resulting in death of parasite (Pettersen et al., 2004). Artemisinin are activated by binding of Fe^2+^ of heme/non-heme and generate carbon centered free radicals or reactive oxygen species which cause damage to cellular targets in their vicinity through alkylation (Cui and Su, 2009). Recently, Hartwig et al. have demonstrated that artemisinin accumulates in neutral lipid of parasite membrane and damage it (Price et al., 1996) which was also advocated by Wang et al. who studied that artemisinin depolarize the mitochondrial membrane via ROS generation which lead to the mitochondria malfunctioning and parasite cell death (Amaratunga et al., 2012). On the other hand Gopalakrishnan and his group has revealed that artesunate induces DNA double-strand breaks in *P. falciparum* with simultaneous increase in intercellular ROS level in the parasite ultimately causing parasite death (Phyo et al., 2012). DNA damage caused by ROS generation in ARS treated mice and impaired function of mitochondria in parasite due to artemisinin treatment providing the premise for our hypothesis that in addition to the above-mentioned effects, the antimalarial action of artemisinin derivatives involves apoptotic cell death in parasite.

Before going to check the apoptotic markers, first we have studied the interaction of these artemisinin derivatives; ART/ ARS and 97/78 with metacaspase enzyme by *in silico* study and results of docking studies have shown that these drugs effectively bind with the metacaspase enzyme which was further confirmed by *in vitro* studies. Although the *P. falciparum* genome contain metacaspase instead of classical caspase-3 (PlasmoDB, 2013), the cytosolic fraction of artemisinin derivatives and synthesized 1,2,4 trioxane treated parasites significantly cleaved the caspase-3 substrate, Z-DEVD-R110 whereas the same fraction of control parasites (untreated) showed very little activity (Figure 3). Moreover in the presence of caspase-3 inhibitor, caspase activity found to be decreased. Finding of *in silico* and *in vitro* studies indicate that parasite's metacaspase has caspase 3 like properties. Except the caspase activation, other markers of apoptotic cell death; mitochondrial membrane potential and DNA fragmentation were also studied to evaluate the apoptotic cell death phenomena in asexual stages of malaria parasite. Depolarization of mitochondrial membrane potential is the characteristic feature observed in cells that are undergoing programmed cell death. In the cells, which are going to apoptotic cell death, mitochondrial membrane potential found to be low which allow release of cytochrome c in cytoplasm. Cytoplasm activate caspases followed by apoptosome formation and cell death (Gottlieb et al., 2003). Here we observed that mitochondrial membrane potential of malaria parasites was getting reduced in the presence of artemisinin derivatives and CDRI 97/78. Our results showed a significant decrease in JC-1 positive cells which indicate the low mitochondrial membrane potential in drug treated parasites as compared to untreated parasites which advocate the findings of Wang and his coauthors (Wang et al., 2010) and also indicate that mitochondrial machinery of Plasmodium could be the target of artemisinin derivatives and synthetic trioxane. Next, DNA fragmentation was quantified from fixed and isolated parasites by the flow cytometeric TUNEL assay as fragmentation in genomic DNA is considered as the hall-mark of apoptotic cell death. Here we observed increased number of fragmented DNA in drug treated parasites as compare to untreated parasite, which resemble with the finding of A.M Rama Gopalkrishna who had suggested that artesunate induce ROS dependent DNA fragmentation in malaria parasite (Gopalakrishnan and Kumar, 2014; Figure 5). These findings for arteether/CDRI 97/78 are reported for the first time. Since the mechanism of PCD induction usually includes an increase in the levels of reactive oxygen species (ROS). Monitoring of oxidative burst revealed significant increase in generation of reactive oxygen species (ROS) in ART, ARS, and 97/78 treated parasites after 24 h (*P* < 0.01) (Figure 6). These findings indicate that artemisinin derivatives induce free radicals mediated apoptotic cell death in asexual stages of malaria parasite.

**Figure 7:**
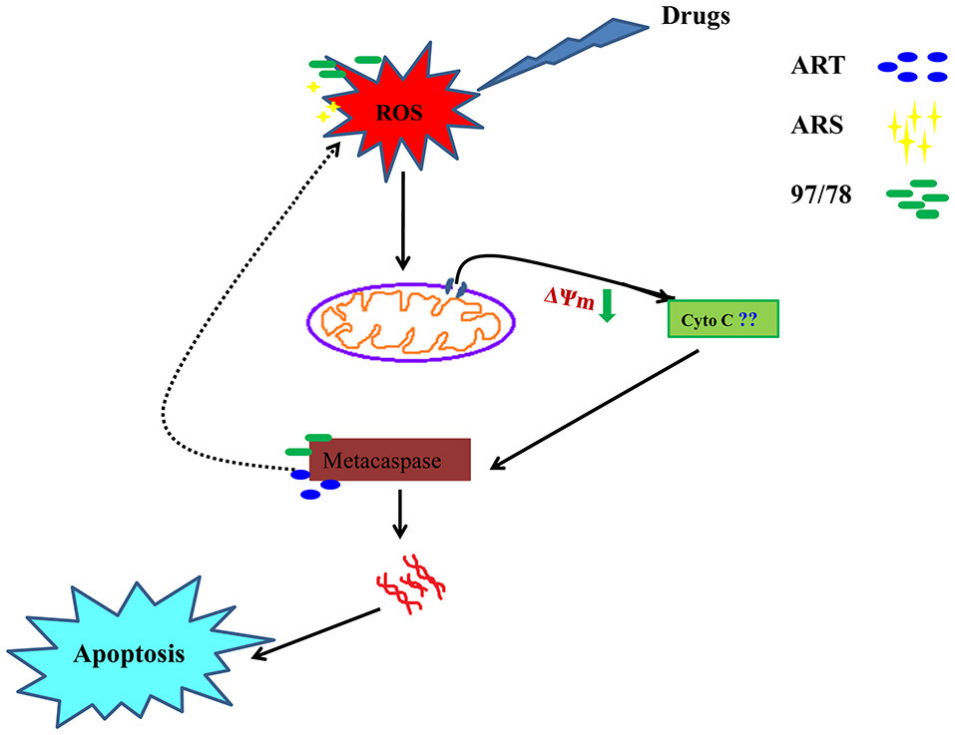
A systematic flow diagram representing action mechanism of artemisinin derivatives and 1,2,4 trioxane via apoptotic pathway. Cleavage of endoperoxide bridge of these drugs generate ROS and further permeabilization of mitochondrial membrane occur followed by activation of metacaspase. Activated caspase like enzyme start to degrade proteins and free to DNase.

In the present study we have concluded that these drugs generate ROS due to the cleavage of endoperoxide bridge which cause depolarization of mitochondrial outer membrane and lead to its permeabilization. Further caspase like enzyme get activated and start degradation of different proteins and free to DNase. In higher eukaryotes, cytochrome c (Cyt C) release from mitochondria and induce the activation of caspases but in Plasmodium, role of cytochrome c in apoptosis is still unknown so here we have hypothesized that may be Cyt C or Cyt C like any other protein could be involved in the activation of metacaspase (Figure 7).

## Author contributions

SG has helped in designing experiments and analyzing the results of experiments, performed most of the experiments and wrote the manuscript. TS has performed *in silico* experiments of this study. KY has done the experiment to measure the oxidative burst after treatment of drugs. BSC and SKS have helped in doing the experiments to find out antimalarial activity of arteether, artesunate and synthetic trioxane CDRI-97/78. MIS designed the *in silico* experiments and analyzed the results of computational studies. RT has designed the whole study as well as objectives and analyzed all results/outcomes of each experiment and also helped in writing manuscript.

### Conflict of interest statement

The authors declare that the research was conducted in the absence of any commercial or financial relationships that could be construed as a potential conflict of interest.
