# Supplementary material for: Artemisinin Derivatives and Synthetic Trioxane Trigger Apoptotic Cell Death in Asexual Stages of Plasmodium
Source: Front Cell Infect Microbiol. 2018 Jul 26;8:256. doi: 10.3389/fcimb.2018.00256 (PMC6070741; doi:10.3389/fcimb.2018.00256)
Supplement: Supplementary file 1 [file Table_1.docx]

| Sr. No. | Name of the Molecule | Residue(s) involved in interaction | Nature of Bonding |
| --- | --- | --- | --- |
| 1. | Arteether | Ser278 | Hydrogen Bond |
|  |  | Phe290 | Hydrophobic |
|  |  | Gln249, Asn253, His273, Gln276, His277, Asn291, Asn314 | Polar |
|  |  | Lys275, Lys289 | Charged Positive |
| 2. | Artesunate | Asn253,Asn279 | Hydrogen Bond |
|  |  | Gln249, His273, Asn314 | Polar |
|  |  | Lys289 | Charged Positive |
|  |  | Phe290 | Hydrophobic |
| 3. | 97-98 parent compound | The245, Ser278, Asn279, Asn291 | Polar |
|  |  | Pro239, Val248 | Hydrophobic |
|  |  | Gly240, Gln249, Lys289, Asn314 | Hydrogen Bond |
|  |  | Phe290 | Pi-Pi interaction |
|  |  | Lys252 | Charged Positive |

**Table S1: Important residues involved in interaction and their nature of bonding**
